# Spatial memory and learning: investigating the role of dynamic visual acuity

**DOI:** 10.3389/fnbeh.2024.1429069

**Published:** 2024-08-29

**Authors:** Burak Kabiş, Emre Gürses, Ayşe Ýlksen Çolpak Işıkay, Songül Aksoy

**Affiliations:** ^1^Department of Audiology, Faculty of Health Science, Gazi University, Ankara, Turkey; ^2^Department of Audiology, Faculty of Health Science, Hacettepe University, Ankara, Turkey; ^3^Department of Neurology, Faculty of Medicine, Hacettepe University, Ankara, Turkey; ^4^Department of Audiology, Faculty of Health Science, Lokman Hekim University, Ankara, Turkey

**Keywords:** virtual Morris Water Maze Test, dynamic visual acuity, spatial learning, spatial memory, trail-making test

## Abstract

**Introduction:**

The vestibular system’s contribution to spatial learning and memory abilities may be clarified using the virtual Morris Water Maze Task (vMWMT). This is important because of the connections between the vestibular system and the hippocampus area. However, there is ongoing debate over the role of the vestibular system in developing spatial abilities. This study aimed to evaluate the relationship between Dynamic Visual Acuity (DVA) across three planes and spatial abilities.

**Methods:**

This cross-sectional study was conducted with 50 healthy adults aged 18 to 55 with normal stress levels and mental health and no neurological, audiological, or vestibular complaints. The Trail-Making Test (TMT) Forms A and B for the assessment of executive functions, the DVA test battery for the evaluation of visual motor functions, and the Virtual Morris Water Maze Test (vMWMT) for the assessment of spatial learning and spatial memory were performed. All participants also underwent the Benton Face Recognition Test (BFRT) and Digit Symbol Substitution Tests (DSST) to assess their relation with spatial memory.

**Results:**

DVA values in horizontal (H-DVA), vertical (V-DVA), and sagittal (S-DVA) planes ranged from (−0.26) to 0.36 logMAR, (−0.20) to 0.36 logMAR, and (−0.28) to 0.33 logMAR, respectively. The latency of three planes of DVA was affected by vMWMT (Horizontal, Vertical, and Sagittal; *Estimate*: 22.733, 18.787, 13.341, respectively *p* < 0.001). Moreover, a moderately significant correlation was also found, with a value of 0.571 between the Virtual MWM test and BFRT and a value of 0.539 between the DSST (*p* < 0.001).

**Conclusion:**

Spatial abilities in healthy adults were significantly influenced by dynamic visual functions across horizontal, vertical, and sagittal planes. These findings are expected to trigger essential discussions about the mechanisms that connect the vestibular-visual system to the hippocampus. The original vMWMT protocol is likely to serve as a model for future studies utilizing this technology.

## Introduction

The vestibular system is a crucial control center in advanced mammals. Although this system is mainly involved by the vestibular ocular reflex (VOR), which is used for rapid action against falls, the association of the vestibular system with cognitive performance has become an essential issue of discussion among researchers (Hitier et al., 2014). These connections regulate self-motion perception, bodily self-consciousness, spatial navigation, spatial learning, spatial memory, and object recognition memory. However, evidence about the connection between dynamic visual acuity and spatial abilities needs to be more extensive (Hitier et al., 2014).

It is commonly known that VOR and visual acuity are related (Kingma and Van de Berg, 2016). Visual acuity refers to the spatial resolution mechanism of the visual system and typically refers to the clarity of vision when both the observer and the target are stationary. Dynamic visual acuity (DVA) is a test where visual acuity is assessed while the head moves, in contrast to static visual acuity, which is evaluated with the head stationary. It is the ability to resolve spatial details of a visible object or image during motion and is an indirect determinant of the VOR function (Schubert et al., 2008).

In the last two decades, the association of the vestibular system with cognitive performance, especially spatial memory, has become an essential issue of discussion among researchers (Hanes and McCollum, 2006). The vestibular system provides the human brain with up-to-date information about head position and visuospatial perceptions. Visual-spatial perception describes how the brain perceives two or three-dimensional spaces and organizes this information. It includes skills such as visuospatial perception, spatial memory, rotation, distance perception, depth perception, and navigation (Bigelow and Agrawal, 2015). Spatial memory comprises a complex structure containing information about various elements of their surroundings, including geometrical design, position, distance, size, direction, and coordinates (Bigelow and Agrawal, 2015; Yoder and Taube, 2014).

More than a century has passed since the first studies on spatial learning and spatial memory. Willard Small (1901) created a labyrinth model as a maze system to study spatial abilities in rats (Wijnen et al., 2024). Numerous researchers have developed different perspectives based on this concept, and Tolman (1949) first mentioned cognitive map theory (Tolman, 1949). The hippocampus was recognized as a critical structure for spatial learning and spatial memory skills at the end of the 20th century (Eichenbaum and Cohen, 2001). The hippocampus and parahippocampal areas receive projections from the vestibular nuclei, and these regions are essential for navigation and spatial memory. These nuclei, which receive information about head motions and spatial orientation from the inner ear, are essential to the vestibular system. They combine this sensory data and send it to the cerebral cortex, thalamus, cerebellum, and other brain areas. The hippocampus uses this vestibular information to create and maintain cognitive maps of its surroundings (Barmack and Yakhnitsa, 2021; Shaikh et al., 2013). It was observed that the dorsal medial temporal areas, which respond to visual signals, were activated by inertial motions. In contrast, no activation was observed in animals with bilateral vestibular disorders in the study on the role of the vestibular system in spatial cognition in primates (Brandt et al., 2005). Previous experimental studies with rats also stated that the vestibular system is crucial for accurate spatial performance, and visual cues alone are insufficient (Thornberry et al., 2021). Due to technological advancements, different assessment techniques have been created that enable the study of spatial learning and spatial memory in humans. Morris (1984) developed the Morris Water Maze Test to assess spatial abilities in rats, and it has been widely considered in the literature as the gold standard test for testing spatial cognition in rodents (Morris, 1984). The virtual Morris Water Maze Test (vMWMT) protocol was inspired by this and was developed for humans for use in computer and virtual reality systems (Hamilton et al., 2002).

Considering the vestibular system’s connections with the hippocampal area, vMWMT may play an essential role in clarifying the vestibular system’s involvement in spatial learning and spatial memory skills. However, the vestibular system’s role in forming spatial abilities is still debatable. Our objective is to assess the correlation between Dynamic Visual Acuity (DVA), which examines the functional status of the vestibular system across three planes, and the findings from vMWMT, which evaluates spatial learning and memory abilities.

## Materials and methods

This study aimed to investigate the effects of ocular motor abilities on spatial learning and spatial memory in healthy adults. It was carried out with the ethical committee’s approval (GO 20/752) by Hacettepe University Non-Interventional Clinical Research Ethics Committee.

### Participants

Participants, fifty healthy adults (Female: 27, Male: 23) between the ages of 18 and 55 ($\bar{\text{X}}$ ± SD: 35.22 ± 10.57), had Mini-Mental State Test score of 24 or higher, a score of less than 40 on the Statement and Trait Anxiety Scale, no known hearing or balance problems, no known neurological problems, at least a primary school graduate, no known head trauma, and no history of surgery were included in the study. Excluded criteria were participants with cervical spine issues, vision problems that cannot be resolved with visual aids (glasses, contacts, etc.), and a history of using drugs that may affect the results of a study, such as antidepressants and sedatives. Each participant had to sign the informed consent form.

### Demographic information

Fourty-two of the participants had right-handed, and eight had left-handed dominance. The body mass indexes (BMI) of the participants were grouped as underweight, normal, pre-obese, and obese according to WHO criteria (Report of a Who consultation, 2000). None of the participants had a virtual reality experience. The demographic information of the participants is shown in Tables 1, 2 in detail.

**TABLE 1:** Demographic information about age, BMI, and mental and anxiety state of participants.

Demographic Information-1	$\bar{\text{X}}$ ± SD	Median	Min-Max
Age (years)	35.22 ± 10.57	33.5	18–55
18–39 (n: 33)	29.00 ± 6.08	30	18–39
40–55 (n: 17)	47.29 ± 5.84	45	40–55
BMI (m/kg)	26.27 ± 4.42	26.28	17.72–37.18
Underweight (n:1)	17.72	17.72	–
Normal (n:20)	22.42 ± 1.79	22.16	19.03–24.68
Pre-Obesity (n:22)	27.77 ± 1.47	27.70	25.31–29.76
Obesity (n:7)	33.72 ± 2.75	34.31	30.07–37.18
MMSE	27.88 ± 2.15	28	24–30
SAS	22.24 ± 2.49	22	20–29
TAS	27.06 ± 7.59	28	21–36
BFRT	48,4 ± 4,12	50	39–54
TMT Form-A	15,94 ± 5,89	15,28	9,17–33,21
Form-B	30,16 ± 14,24	24,43	13,42–75,48
DSST	46,38 ± 15,05	51	15–66

$\bar{\text{X}}$: Mean, SD: Standart Deviation, m: Meters, kg: Kilogram, n: Number of participants, MMSE: Mini-Mental State Examination, SAS: State Anxiety Scale, TAS: Trait Anxiety Scale, BMI: Body Mass Index, Underweight: *BMI* < 18,50; Normal: 18,50 ≤ *BMI* ≤ 24,99: Pre-Obesity: 25,00 ≤ *BMI* ≤ 29,99; Obesity: *BMI* ≥ 30, Min: Minimum value, Max: Maximum Value.

**TABLE 2:** Demographic information about gender, hand dominance, education, eye color, vision problem/using glasses or contact lenses, daily smartphone/tablet/computer usage, game experience, daily duration of sleep, tobacco and alcohol, interest in sports, use of the navigational tool or application, ability to locate.

Demographic Information-2			n	n (%)	N
Gender	Female		27	54%	50
	Male		23	46%	
Hand Dominance	Right		42	84%	50
	Left		8	16%	
Education	Pre-School		8	16%	50
	High School		19	38%	
	University		11	22%	
	Post-Graduate		12	24%	
Eye Color	Brown		18	36%	50
	Blue		9	18%	
	Green		10	20%	
	Hazel		13	26%	
Vision Problem/Using Glasses or Contact Lens	No problem		42	84%	50
	Myopia	Glasses	4	8%	
		Contact Lens	4	8%	
Daily Smartphone, Tablet, and Computer Usage	0–60 min.		9	18%	50
	61–180 min.		11	22%
	181–300 min.		13	13%
	301 min. and above		17	34%
Game Experience	No		32	64%	50
	yes		18	36%	
Daily duration of sleep	0–300 min.		14	28%	50
	301–360 min.		10	20%	
	361–420 min.		11	22%	
	421 min. and above		15	30%	
Tobacco	Yes		25	50%	50
	No		25	50%	
Alcohol	No		16	32%	50
	Rarely		16	32%	
	Sometimes		10	20%	
	Often		7	14%	
	Very Often		1	2%	
Interest in sports	Not Interested		35	70%	50
	Amateur		12	24%
	Professional		3	6%
Use of the navigational tool or application	No		11	22%	50
	Rarely		5	10%	
	Sometimes		12	24%	
	Often		11	22%	
	Very Often		11	22%	
Ability to locate	Yes		22	44%	50
	No		14	28%	
	Sometimes		14	28%	

### Protocols

Mini-Mental State Examination (MMSE) and State and Trait Anxiety Inventory (STAI-TX) with Turkish normalization forms were used to exclude the presence of psychological effects of mental problems. The Digit Symbol Substitution Test (DSST) to assess visuospatial memory skills, Benton Face Recognition Test-Short Form (BFRT-SF) to evaluate visual memory and occipital lobe function, the Trail Making Test (TMT Form-A and Form-B) to assess executive functions, the Dynamic Visual Acuity (DVA) Test to evaluate visual motor skills, and the virtual Morris Water Maze Test (vMWMT) to assess spatial learning and spatial memory were used. The scales were the main tools used in the evaluation processes. Each participant was applied to TMT and DVA, respectively, and asked to take a 30-min rest. In the last part, virtual MWMT was done. It has been calculated that it will take approximately 105 min to administer the entire test protocol to one participant.

### Mini-mental state examination (MMSE)

MMSE, developed by Folstein et al. (1975), is a widely used test in clinical practice to detect and follow cognitive impairment (Folstein et al., 1975). Five categories comprise the MMSE, which assigns a total of 30 points for evaluation: orientation (10 points), recording memory (3 points), attention and computation (5 points), recall (3 points), and language abilities (9 points). According to the Turkish validity and reliability study by Güngen et al. (2002), a test score of 24 or more points is normal.

### State and trait anxiety scale (STAI-TX)

STAI-TX was developed by Spielberger (1985) and adapted for Turkish by Le Compte and Oner (1976). It is a 40-question scale with 20 questions and two parts for each (State and Trait). In the State Anxiety Scale (SAS), participants are asked to describe their feelings under certain circumstances at a specific time and respond to this section while considering their current context. The Trait Anxiety Scale (TAS) evaluates a person’s level of anxiety regardless of their environment or circumstances.

In both parts of the scale, there are two types of expressions: expressions showing positive emotions (direct) and words showing negative emotions (reverse). When scoring, the total weighted score of the questions with direct statements is deducted from the total weighted score of the questions with opposing statements. This number is then added to the set value of 50 for SAS and 35 for TAS, resulting in the person’s anxiety score. Individuals with scores of 40 or lower were included in the study.

### Trail making test (TMT)

The Trail Making Test measures executive functioning, working memory, complex attention, motor skills, and visuospatial and motor skills. TMT is divided into two sections: “Form-A,” which assesses the processing speed of visual scanning abilities, and “Form-B,” which assesses the capacity to switch the setup between stimulus sets and maintain the order. The Turkish version of TMT was used (Cangöz et al., 2007), with no fixed time limit for finishing the test. Participants were expected to complete the test, and statistical analyses were solely conducted on the Form-A completion time (FAT) and Form-B completion time (FBT) values.

### Digit symbol substitution test (DSST)

DSST is one of the subtests of the Wechsler Adult Intelligence Scale. It is used as a neuropsychological test to evaluate sensory-motor processing, visual perception, scanning abilities, and visuospatial memory abilities (Jaeger, 2018). In the DSST, the participant is given 90 seconds to fill the boxes with the numbers corresponding to the symbols. The boxes that need to be checked are shown with symbols for 1 through 9. The Turkish version (Ozakbas et al., 2017) was used in our study despite the claim that genetic characteristics, gender, culture, language, and education had a minimal effect.

### Benton Face Recognition Test-Short Form (BFRT-SF)

BFRT-SF (Benton and Van Allen, 1968), Turkish validity, and reliability were conducted by Keskinkiliç (Keskinkiliç, 2008). The BFRT-SF is a 13-page, A-4-sized, spiral-bound book containing facial pictures that ask for replies on a form. The test that evaluates facial recognition abilities and assesses visuospatial perception is called an evaluation of occipitotemporal lobe functions.

### Dynamic visual acuity (DVA) protocol

**FIGURE 1:**
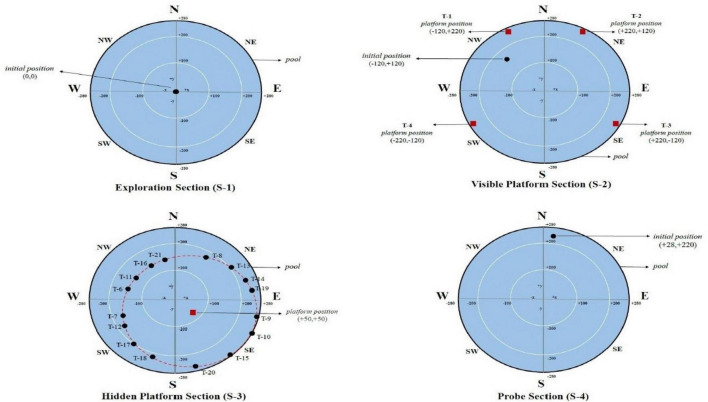
Initial and platform positions of the participants in the exploration section (S-1), the visible platform section (S-2), the hidden platform section (S-3), and the probe section (S-4) in the vMWM test protocol are shown in pool diagrams. T: Trial, N: North, S: South, W: West, E: East, NW: Northwest, NE: Northeast, SW: Southwest, SE: Southeast.

The Neurocom SMART EquiTest with the InVision software package (version 8.4.0) (NeuroCom, a Division of Natus, Clackamas, OR, U.S.A.) was used for DVA test protocols. Participants were instructed to align their gaze with the test screen while seated in an adjustable-height chair 250 cm from the tool screen. In addition, the participant was asked to indicate the direction of the optotype displayed on the screen when the head was fixed or moving, and the participant’s expressed order of the optotype was marked by the practitioner using a remote control device. The direction of the optotype is the side that the letter (E) opening is towards, and the opening side will be displayed in 4 different directions: right, left, up, and down (Figure 1). This was communicated to the participants.

The subjects performed four different tests from the DVA test battery: the static visual acuity (SVA), minimum perception time (MPT), head motion applied, and the gaze stabilization test (GST) and dynamic visual acuity (DVA), in that order. The ‘Exercise’ module offered by the device was utilized to help the participants better consolidate the test after the application method for each test had been explained to the patients. A device to measure the head movement speed, comprising a speed-sensitive sensor, was placed on the participants’ heads in the GST and DVA, where the head was applied and fixed to the head of the participants to prevent them from slipping during head movements.

The smallest figure size optotype value for which the participant correctly responded at least three out of five trials without head movement is calculated in logMAR and represents the SVA value. Also, Participants in MPT can evaluate the least amount of time to perceive the position of the presented optotype appropriately. While the head is stationary, it is asked to indicate which direction the optotype displayed on the screen can detect. The optotype size is fixed at 0.2 logMAR over the SVA value. The MPT value, which is determined by calculating the screen time in milliseconds (ms), is the one where at least three corrects of the five possible responses. In the GST, participants’ ability to detect the direction of an optotype at the fastest possible head speed is tested. The optotype size was set to be 0.2 logMAR greater than the SVA value. When nodding, numerical and visual representations of the participant’s head’s speed and angular value are displayed on the screen. The participant’s following step was to take speed parameter changes based on how accurately they answered the tasks. If they respond accurately, the speed value increases; if they respond incorrectly, it drops. As a result, the maximum speed at which the person performs gaze stability is determined. By averaging three different head speeds with accurate responses, the GST value was calculated in °/sec. Three various head movements were used: horizontal, vertical, and sagittal. Participants were asked to shake their heads in the method implemented with a speed of 85 /sec and 20 times. Once the required angle and speed value have been accomplished, the system displays the optotype to the participant on the screen. The optotype will not be displayed on the screen if the head speed is insufficient. Instead, the participant will see a warning message and repeat this step. The smallest optotype size, which both the right and left sides correctly identified in 3 out of 5 trials, is used to calculate the DVA value in logMAR. Three different head movements were used: horizontal, vertical, and sagittal (Ward et al., 2010).

### Virtual morris water maze test (virtual MWM Test) protocol

All Virtual MWM test procedures were done with an Oculus Rift (Facebook Technologies, USA) virtual reality glasses integrated with a notebook. The glasses are designed with specialized software and techniques to create the sensation of being in different environments and provide a sense of space and depth. It is composed of cameras and sensors. To ensure the highest level of visual field, the device features a special optical system and supports a minimum resolution of 1920x1080, allowing for at least a 90° peripheral viewing angle. In addition, two external sensors are responsible for transferring the user’s movements to the virtual reality system to detect the user’s movements. These sensors are accompanied by a pair of controllers that provide an intuitive hand experience in virtual reality, allowing the user to feel and use their virtual hands as if they were their own. A pool with a diameter of 560 virtual meters (*v*m) is present in the Virtual MWM test. This pool is surrounded by four walls with cues: a triangle in the north, a circle in the south, a heart in the west, and a square in the east. All of these cues had the same color and were black.

The Virtual MWM test protocol had a total of 22 trials. It was divided into four sections: exploration (Section 1 = one trial), visible platform (Section 2 = four trials), hidden platform (Section 3 = sixteen trials), and probe (Section 4 = one trial).

The exploration section (S 1) lasts 60 seconds and is used to provide participants control while wearing VR glasses, help them get used to moving around in the virtual environment, and help them figure out where various cues on the pool and the surrounding wall are located (Figure 1).

In the visible platform section (S 2), it was asked to get to the red square platform, which was set up and visible from various points around the pool during the four trials, each with a time limit of no more than 60 seconds. There was a 10-second rest interval between each trial. In each motor control segment trial, the platform positions’ coordinates are shown in Figure 1.

Spatial learning performance is evaluated in the hidden platform section (S 3). Participants in each trial began the test in a different location, the coordinates of which had been chosen on the virtual pool by the researcher. Participants were instructed that there was a hidden platform at any point in the pool, that they had to find it, and that each trial had 60 sec. and a maximum of 16 trials to find it. If they found it within sixteen attempts, they were asked to remember it again and to find the spot where the hidden platform was until the end of this section. There was a 10 sec—rest interval between each trial. Sixteen trials were analyzed as four blocks. Therefore, in this section, the initial coordinates of the participants were equally distributed to the pool quarters using the Latin Square method (Newhouse et al., 2007). Figure 1 shows the initial and hidden platforms’ position on the task’s pool diagram in each trial.

The probe section (S 4) evaluates spatial memory abilities. This section was started after a 10-min rest period following the end of the hidden platform section. The platform was entirely removed from the pool in this section (Figure 1). However, the participants were instructed that it was still in the same position, and they had 60 seconds to find it again. Instead of trying to find the platform, this section aimed to determine how much time the participants spent in the pool during 60 seconds when they were closest to it (in the pool quadrant where the platform is located). In the Virtual MWM test, the outputs include how much time and distance each participant spent in the quadrant of the pool. The ability of spatial memory was therefore assessed using the preference score (Gonzalez et al., 2000). The search time in the platform quadrant (P) was subtracted from the search time in the non-platform quadrants (A, B, and C) to get this score: Preference Score (PS): $\frac{\left[\left(P-A\right)+\left(P-B\right)+\left(P-C\right)\right]}{3}$.

### Data analysis

The study was analyzed using SPSS 23.0 (IBM, Corp, NY, USA) and R (R Core Team, Vienna, Austria) statistical package software programs. For quantitative data from descriptive statistics, mean (X), standard deviation (SD), median, and minimum (min.)-maximum (max.) value expressions were used, whereas numbers (n) and percentages (%) were employed for qualitative data. To evaluate whether there are any differences in demographics between groups, the Student-t test was used between paired groups with parametrically distributed data, and Post Hoc tests (homogeneous distribution of variance, Bonferroni test, inhomogeneous distribution of variance, Tamhane’s T2 test) were used for pairwise comparisons within multiple groups. The Mann-Whitney U test was used between two groups that did not show the parametric distribution, and the Kruskal-Wallis test was used for comparisons of more than two groups. The difference between multiple repeated measurements and demographic data was also evaluated using the repeated measures ANOVA (rmANOVA) test. The effects of demographic data (age, BMI, etc.) and data of DVA on VMWMT parameters were assessed using multiple regression models. Using graphs (histogram and Q-Q plot) and regular distribution tests, the residuals of the regression model were found to follow the normal distribution. In the established models, the Durbin-Watson value was between 1.5 and 2.5 to rule out autocorrelation, and the VIF value was less than 5 to rule out variable variance and multicollinearity. The stepwise method was used to design multiple regression models that fulfilled these criteria and were statistically significant. Marginal models were created to model the variation of the parameters of the DVA test battery in repeated measurements. For marginal model analysis, the “gee” package in the R program was used (Carey et al., 2012). The accepted statistical significance level was *p* < 0.05. Sample size estimation was calculated using G*Power version 3.1.6. In addition, the study by Brainbouer et al. (Breinbauer et al., 2019) that examined spatial navigation in vestibular disorders served as a guide in determining the appropriate sample size; the alpha was 0.05, the beta was 0.05, with a 95% power, and the number of individuals to be included in the study was calculated as 48.

## Results

### Neuropsychologic outcomes

The scores of the participants for BFRT, DSST, and TMT are shown in Table 1. Accordingly, the mean BFRT score is 48.4 ± 4.12 (range 39–54), the mean DSST score is 46.38 ± 15.05 (range 15–66), the mean FAT is 15.94 ± 5.89 (range 9.17–33.21 sec), and the mean FBT; 30.16 ± 14.24 (range 13.42–75.48 sec) were found.

### DVA Outcomes

The values for SVA and MPT ranged between (−0.32)–0.48 logMAR and 10–80 msec, respectively. GST, H-GS, V-GS, and S-GS values ranged between 93 and 198, 5°/sec, 84.5–201°/sec, and 86.5–160°/sec, respectively (Table 3). In DVA, H-DVA, V-DVA, and S-DVA values, they ranged between (−0.26)–0.36 logMAR, (−0.20)–0.36 logMAR and (−0.28)–0.33 logMAR respectively. Between the right and left sides in the horizontal plane, the ups and downsides in the vertical plane, and the right and left sides in the sagittal plane, there was no statistically significant difference for GST and DVA (*p* > 0.05). The head movement plan also showed no statistically significant difference between the horizontal, vertical, and sagittal planes (*p* > 0.05). Therefore, by averaging the used sides (right-left, up-down) within the planes, data corresponding to planes (horizontal-vertical-sagittal) were used in multiple regression and marginal model analysis (Table 3).

**TABLE 3:** SVA, MPT, GST, and DVA findings in the horizontal, vertical, and sagittal planes of the participants.

DVA Protocols		$\bar{\text{X}}$ ± SD	Median	Min-Max	*p* ^a^	*p* ^b^
**SVA (logMAR)**		(−0.05) ± 0.22	−0.09	(−0.32)−0.48	–	–
**MPT (msec)**		31.00 ± 12.49	30	10–80		–
GST (°/sn)	H-R	133.30 ± 22.74	132	88–205	0.781	–
	H-L	130.50 ± 20.83	130.5	95–192		
	V-U	118.56 ± 25.03	117	85–188	0.719	
	V-D	121.06 ± 24.66	120.5	80–217		
	S-R	124.76 ± 16.73	126	83–161	0.449	
	S-L	123.62 ± 19.03	122.5	84–175		
	*HM.*	131.90 ± 21.39	131	93–198.5	–	0.615
	*VM*	119.81 ± 23.99	120	84.5–201		
	*SM*	124.19 ± 17.09	123.5	86.5–160		
DVA (logMAR)	H-R	0.07 ± 0.18	0.10	(−0.28)–0.36	0.649	–
	H-L	0.07 ± 0.17	0.11	(−0.24)–0.36		
	V-U	0.10 ± 0.16	0.12	(−0.20)–0.32	0.436	
	V-D	0.11 ± 0.18	0.14	(−0.20)–0.40		
	S-R	0.11 ± 0.17	0.18	(−0.30)–0.36	0.615	
	S-L	0.12 ± 0.17	0.20	(−0.26)–0.32		
	*HM.*	0.07 ± 0.17	0.07	(−0.26)–0.36	–	0.231
	*VM*	0.11 ± 0.16	0.11	(−0.20)–0.36		
	*SM*	0.12 ± 0.17	0.17	(−0.28)–0.33		

DVA, Dynamic Visual Acuity; SVA, Static Visual Acuity; H, Horizontal; V, Vertical; S, Sagital; R, Right; L, Left; U, Up; D, Down MPT, Minimum Perception Time; GST, Gaze Stabilization Test; HM, Mean of right and left direction values in the horizontal plane; VM, Mean of up and down direction values in the vertical plane; SM, Mean of right and left direction values in the sagital plane; $\bar{\text{X}}$, Mean; SD, Standard Deviation; msec, miliseconds. *p*^a^: Mann Whitney U Test, *p*^b^: Kruscal Wallis Test, *p* < 0.05: Statistically significant.

### Virtual MWM test outcomes

In 60 seconds, the participants traveled an average path length of 1.24 ± 0.32. No statistically significant difference regarding gender, age, or other indicated demographics was found in the S1 (*p* > 0.05). Additionally, there was no statistically significant difference between the participants’ mean path length and mean latency and demographics for the four trials in S2 (*p* > 0.05).

In S-3, latency and path length were analyzed by splitting the 16 trials into four groups and using their averages. Accordingly, the calculated mean latency and path length values for Trials 6, 7, 8, and 9 (T6–9) were 49.58 ± 8.41 sec and 1.28 ± 0.24, respectively. The mean path length and latency for trials 10, 11,12, and 13 (T10–13) were 23.69 ± 14.63 sec and 0.58 ± 0.31, respectively. The mean path length and latency for trials 14, 15, 16, and 17 (T14–17) were 27.95 ± 11.18 sec and 0.69 ± 0.18, respectively. Lastly, the mean path length and latency for trials 18, 19, 20, and 21 (T18–21) were 13.29 ± 7.81 sec and 0.31 ± 0.11, respectively.

**FIGURE 2:**
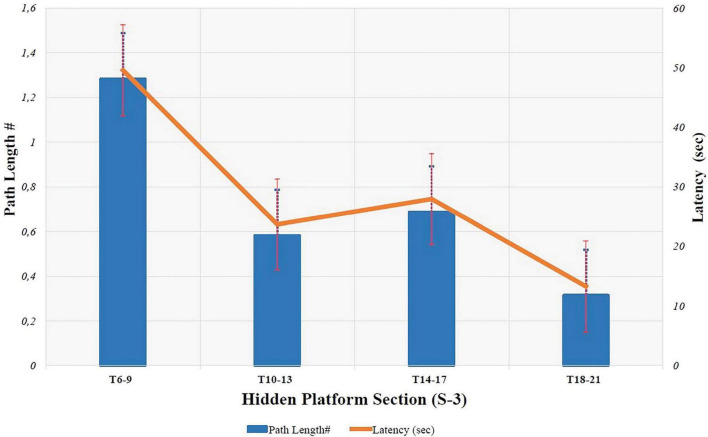
Path length and latency graph of 16 trials analyzed in 4 blocks in the hidden platform section (S-3). T: Trial, Sec: Seconds, #: represents the ratio of the path length to the pool’s diameter.

*rm*ANOVA was used to evaluate whether there was a statistically significant difference between the four groups (T6–9, T10–13, T14–17, and T18–21). Accordingly, a statistically significant difference between the groups was found [path length: *F* (3.109) = 214, *p* < 0.001 and latency: *F*(3.116) = 243, *p* < 0.001]. It indicates that the participants had achieved spatial learning. However, there was a statistically significant difference in pairwise comparisons (*p* < 0.001), although no statistically significant difference was found between T10-13 and T14-17 (*p* > 0.05). The findings consistently indicated that most participants in the first four sections had not yet found the hidden platform. However, the sections in which participant spatial learning abilities varied were between the 10th and 17th trials. Figure 2 shows the path length and latency graph for the four groups in S-3. Additionally, in the hidden platform section, there were no statistically significant differences in *rm*ANOVA in terms of both path length and latency between demographics such as gender, hand dominance, daily smartphone/tablet/computer usage, eye color BMI, daily duration of sleep, alcohol, vision problem /using glasses or contact lenses, interest in sports, and game experience (*p* > 0.05). Age-based analysis, however, showed a statistically significant difference between the age groups [path length: *F* (3.144) = 5.7; *η^2^* = 0.146; *p* = 0.008 and latency: *F* (3.144) = 6, *η^2^* = 0.139; *p* < 0.001]. Only the T10-13 and T14-17 trials revealed this difference between age groups. It was, therefore, believed that participants over the age of 40 had a negative impact on their spatial learning abilities. Furthermore, analysis of the effects of smoking showed a statistically significant impact on repeated assessments of spatial learning [path length and latency: *F* (3.144) = 5, *η^2^_*pathlenght*_* = 0.144, *η^2^_*larency*_* = 0.131; *p* < 0.01]. It was found that this parameter only affected the T10-13 and T14-17 trials, similar to the age analysis. These findings consistently indicated that smoking had a negative effect on spatial learning abilities.

In S-4, the preference score (PS) was calculated based on the path length by the participants in 60 seconds and the time spent in the platform quarter. Accordingly, the mean path length by the participants was 1.64 ± 0.24, and the mean PS was 45.89 ± 5.03). When the mean path length in this trial (T-22) was analyzed in terms of demographics, no statistically significant difference was found (*p* > 0.05). However, when analyzed according to PS, statistically significant differences were found in age (Kruskal-Wallis; *p* = 0.020), education (pre-school/post-graduate: Kruskal-Wallis; *p* = 0.006), vision problem/using glasses or contact lenses (no problem-glasses: Kruskal-Wallis; p = 0.006) and tobacco (Mann Whitney U test; *p* = 0,009). The findings indicated that spatial memory abilities started to decline around the age of 40. Although it was not found to be parallel to the increase in education level, it was believed that advanced academic level positively improved spatial memory abilities. Additionally, the fact that the mean PS values of the contact lens users were similar to those of non-users and that there was no difference between them and those without impaired vision indicates that test cooperation had been affected by the usage of glasses rather than spatial memory abilities. Lastly, it was also found that tobacco might be detrimental to spatial memory.

### DVA and virtual MWM test

Multiple linear regression was used to examine the interaction between visual motor function and spatial memory abilities. In contrast, repeated measurements used marginal models to analyze the link between visual motor function and spatial learning abilities (path length and latency). The stepwise method was used to create models between DVA parameters and spatial skills in addition to demographics.

**FIGURE 3:**
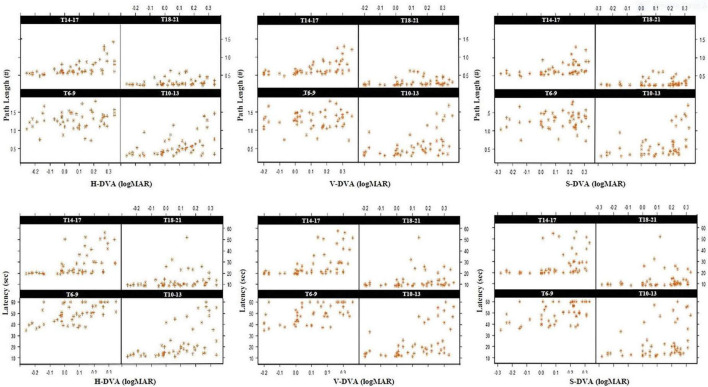
Standardized scatter plot graphs of marginal model analysis in repeated measurements established between horizontal, vertical, and sagittal DVA and path and time values of the hidden platform section (S-3) assessed in 4 blocks (T6-9, T10-13, T14-17, and T18-21). T: Trial, Sec: Seconds, H: Horizontal, V: Vertical, S: Sagittal, #: represents the ratio of the path length to the diameter of the pool.

**FIGURE 4:**
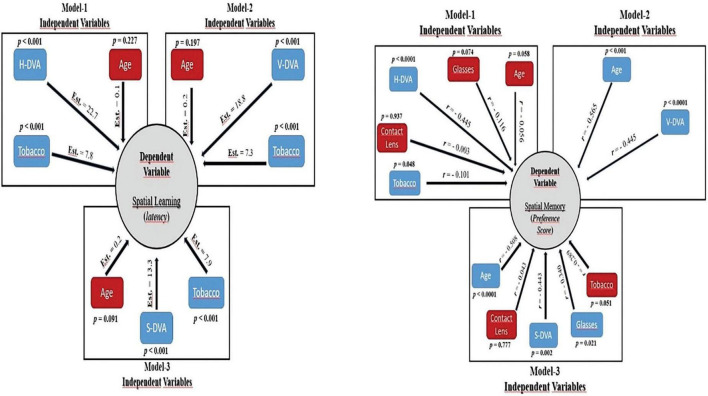
Schematic representation of Marginal model analysis between DVA and spatial learning (left) and multiple regression analysis between DVA and spatial memory (right) with various demographics. *r*: partial correlation, Est: Estimate value, *p* < 0.05: Statistically significant.

Although there were models in which age had a statistically significant effect on models created with SVA, MPT, and demographics, there were no statistically significant models in which spatial learning with SVA, MPT, and GST (for horizontal, vertical, and sagittal) affected on path length and latency (*p* > 0.05). However, there were models with a statistically significant effect on latency in all three planes (Horizontal, Vertical and Sagittal; *Estimate*: 22.733, 18.787, 13.341, respectively *p* < 0.001), while there were no statistically significant models in which spatial learning had an effect on path length in the models created by using horizontal, vertical, and sagittal plane data of DVA (Figure 3). In addition, it was found that tobacco had a statistically significant effect on latency in the models rather than path length (Horizontal, Vertical, and Sagittal; *Estimate*: 7.760, 7.251, 7.974, respectively *p* < 0.001). The dependent variable—the spatial learning parameters—and all the independent factors are schematically depicted in Figure 4.

SVA, MPT, GST, and did have no statistically significant effect on path length and PS of the spatial memory in the models (*p* > 0.05). However, there were statistically significant models between DVA demographics and PS (*p* < 0.05).

The multiple regression analysis using H-DVA, age, tobacco, vision problem /using glasses or contact lenses as the independent variables and PS as the dependent variable showed that 57.6% of the variance in the PS variable was explained by independent variables [*F* (5.44) = 14.324; *p* < 0.0001]. As a result, the PS parameter was significantly and negatively predicted by the H-DVA parameter [β = (−0.502); t(44) = (−4.102); *p* < 0.001; *r* = (−0.445)]. Additionally, tobacco had a significant correlation with the PS variable [β = (−0.198); t(44) = (−2.031); *p* = 0.048; *r* = (−0.101)]. However, it was not found that age and vision problems / using glasses or contact lenses could significantly predict the PS (*p* > 0.05). Furthermore, V-DVA and age as the independent variables and PS as the dependent variable showed that 57.5% of the variance in the PS variable was explained by independent variables [*F*(2.47) = 34.142; *p* < 0.0001]. As a result, the PS parameter was significantly and negatively predicted by the V-DVA parameter [β = (−0.598); t (47) = (−6.104); *p* < 0.001; *r* = (−0.445)]. Additionally, age had a significant correlation with the PS variable [β = (−0.333); t(47) = (−3.404); *p* < 0.0001; *r* = (−0.565)]. S-DVA, age, tobacco, usage of glasses or contact lenses as the independent variables and PS as the dependent variable also showed that 52.9% of the variance in the PS variable was explained by independent variables [*F*(5.44) = 12.012; *p* < 0.0001]. As a result, the PS parameter was significantly and negatively predicted by the S-DVA parameter [β = (−0.359); t (44) = (−3.277); *p* = 0.002; *r* = (−0.443)]. Additionally, age and using glasses had a significant correlation with the PS variable [Age: β = (−0.408); t (44) = (−3.916); *p* < 0.0001; *r* = (−0.508), Using glasses: β = (−0.248); t (44) = (−2.399); *p* = 0.021; *r* = (−0.340)]. However, it was not found that tobacco and contact lenses could significantly predict the PS (*p* > 0.05). The dependent variable—the spatial memory parameter—and all the independent factors are schematically depicted in Figure 3.

## Discussion

Studies on spatial learning and spatial memory with DVA are quite limited in healthy controls and patients with vestibular deficiency. In our research, we found that dynamic visual functions, which are an indirect determinant of vestibular system performance, affect spatial learning and memory, unlike static visual functions. These results are supported by the studies that dynamic visual functions, which require continuous processing and integration of visual stimuli, are significantly influenced by cerebellar activity, thereby impacting spatial abilities more than static visual functions (Rondi-Reig et al., 2022; Koziol et al., 2014).

Patients with vertigo complaints were evaluated with the arithmetic counting back and forth method in the study, and 20% of patients showed cognitive impairment. It was reported that the vestibular system and spatial skills are closely related (Risey and Briner, 1990). The development of experimental methods used on humans has revealed the crucial importance of the hippocampus in processing vestibular information and visual and other sensory information. Studies on vestibular connections with the hippocampus indicate that vestibular system activation may be the primary structure that fires head direction cells in the thalamic regions and place cells in the hippocampus (Barnes et al., 1990; Knierim et al., 1996; McNaughton et al., 1996). The relationship of the vestibular system with spatial memory and spatial learning functions was first reported in humans by Schautzer et al. (2003). The researchers studied with 12 neurofibromatosis type 2 patients with bilateral vestibular neurectomy using the computer-integrated protocol of the Morris Water Maze Test and have reported that chronic vestibular input impairment may cause hippocampal insufficiency (Schautzer et al., 2003). In another study, spatial functions were evaluated with vMWM test protocol and gray matter measurements in the hippocampal and parahippocampal regions with MRI in 15 patients with bilateral vestibular disorders. It was reported that even partial vestibular loss affects spatial learning and spatial memory at anatomical and functional levels (Kremmyda et al., 2016). However, in a study using a protocol similar to our research, patients with bilateral vestibulopathy patients (n = 64) and healthy control (n = 46), it was reported that although bilateral vestibulopathy patients performed worse on vMWMT, spatial memory, and spatial learning functions were not statistically different (Dobbels et al., 2020). These differences might be explained by age, anxiety level of the participants, working memory abilities, and duration and severity of vestibular loss. However, further studies are needed to define clinical factors. The effect of hearing loss was also evaluated, and it was seen that the group with sensorineural hearing loss spent more time finding the hidden platform (Dobbels et al., 2020). In this study with healthy participants, the effect of semicircular canal functions on spatial skills was compatible with the literature. In addition, this study makes an essential contribution to the literature regarding the association between the dynamic visual acuity parameter of the vestibular system function and spatial functions.

Effects of semicircular canal functions on spatial learning and spatial memory, which was one of the main findings of this study, suggests that it may be due to the pathways between the central vestibular system and the hippocampus, which are known among current research topics. It is believed that the cerebellum, which is one of the structures with a critical role in these pathways, receives information from the vestibular nuclei and plays a vital role in the regulation of vestibular reflexes and transforming the egocentric representation to allocentric representation in spatial navigation skills (Dieterich and Brandt, 2015). Studies conducted in experimental animal models (Raudies et al., 2015) and humans (Rochefort et al., 2013) with cerebellar disorders have shown that atrophy and low fever rates are observed in position cells in the hippocampus. According to these findings, dynamic visual functions in all three planes (horizontal, vertical, and sagittal) rather than static visual functions substantially affected spatial abilities, which may result from synaptic connections between the vestibular and spatial pathways in the cerebellum. It is possible to provide a more detailed explanation of the functional outcomes of vestibular-spatial skills at the cerebellar region by combining cerebellum evaluation techniques with the vMWMT and conducting studies on people with both healthy and vestibular disorders. For example, within the video-nystagmography test battery, evaluations of the central vestibular pathways are conducted, providing information about the brainstem and cerebellar functions of the vestibular system through smooth pursuit, saccades, and optokinetic tests (Leigh and Kennard, 2004; Mishra and Singh, 2021). We believe incorporating these methods into spatial skills assessments would contribute significantly to spatial studies.

It is commonly known that spatial navigation skills and vestibular system pathways establish synaptic connections in cortical regions after the cerebellum, and semicircular canal functions are represented as spatial functions thanks to head direction cells. The head direction cells are located in the parahippocampal, entorhinal cortex, thalamus, and retrosplenial cortex (Bellmund et al., 2016). Do et al. (2021) reported that retrosplenial cortex activation occurred with rotational head movements rather than translational head movements. They used EEG during spatial navigation evaluation with a virtual reality system in healthy adults. The authors concluded that the retrosplenial cortex was crucial in calculating the head direction (Do et al., 2021). It was anticipated that these findings would contribute to the literature by clarifying how, depending on the head movement plane, the functions of the head direction cells differ at the cortical level.

Vestibular stimulations that occur with linear and angular movements have an essential role in updating the cognitive map by causing modulation in place cells were reported (Gavrilov et al., 1995). Cuthbert et al. (2000) reported that in experimental animals, electrical and caloric stimulation of the inner ear caused an increase in the activation of place cells in the hippocampal region (Cuthbert et al., 2000). Brandt et al. (2005) reported that bilateral hippocampal atrophy was observed in those with bilateral vestibular disorders, possibly due to the lack of stimulation of place cells (Brandt et al., 2005). In this study’s virtual MWM test protocol, especially in the spatial learning section where sequential assessments were applied, the participants started the test from a different perspective in each trial. With the vMWMT battery, participants employed both egocentric and allocentric strategies. In virtual reality, they tried to find the hidden platform in the trials using head movements at different angles. Although there was a wide variety of elements, it is clear that participants performed the tasks due to the activation of the vestibular system. Therefore, it was considered that the difference between pathological and normal individuals was caused by cells in the hippocampal regions that were particularly placed and the connections in the cortical and thalamic areas. Smooth pursuit and saccadic eye movements, which are among the most essential functions of the central vestibular system and involve the oculomotor connection pathways, are used to provide optimum visual acuity during head movements. Kim et al. (2019) (Kim et al., 2019) examined the association between the oculomotor system and memory with MRI in 145 patients with transient global amnesia with only episodic memory impairment and no other neurological deficits, found lesions in different regions of the hippocampus and impaired smooth pursuit velocity at 10°/s and 20°/s. Tregellas et al. (2004) investigated the neurobiology of smooth pursuit with fMRI in 14 schizophrenic and 14 healthy adults. They reported that people with schizophrenia had increased activation in the posterior hippocampal and right fusiform gyrus regions compared to healthy adults. The saccadic system, a critical neuronal branch of the oculomotor system, is also known to cause modulation in the hippocampus and parahippocampal region in addition to smooth pursuit from previous studies (Hoffman et al., 2013; Jutras et al., 2013). Studies in rodents showed that the hippocampus had low-frequency theta rhythm during sniffing and movement. Studies have established that these rhythms record a previous experience and are used in later recalls. Hoffman et al. (2013) investigated the association between saccadic eye movements and hippocampal theta rhythms based on the theory that saccadic systems also serve this purpose in humans and monkeys. According to their findings, although there was no direct connection between saccadic eye movements and hippocampal low theta rhythm (3–8 Hz), using various methods to analyze the saccadic system may reveal the association. It would not be wrong to claim that the participants used oculomotor eye movements while performing the virtual MWM test protocol. We speculate that the difference in spatial abilities among the participants in this study may be due to the saccadic and smooth tracking connections with the hippocampal regions.

While no effect of GST findings was found in this study, it was determined that DVA affected spatial learning and spatial memory. DVA calculates visual acuity at a constant head speed, whereas GST measures the maximum head speed to maintain visual acuity. Although GST and DVA evaluate the same functions with different parameters, GST is still controversial in the literature because it includes individual differences in visual stimulus sizes, cooperation with varying head speeds, and a logarithmic calculation. Kaufman et al. (2014) reported that in the reliability study of the DVA test protocol, the GST reliability was weak and moderate, and the DVA reliability was moderate and high. The findings from DVA and GST appear more reliable in patients with unilateral vestibular disorders. According to Goebel et al. (2007), GST’s specificity was 93%, and sensitivity was 64% in unilateral vestibular diseases. However, it was reported in another study that the compensatory mechanism that affected the physiology of the VOR could be activated because DVA was applied with a slower head movement than GST (Voelker et al., 2015). The entire visual acuity test battery was used in our research. Because they faced challenges such as cooperation fatigue during the GST, the participants needed more time to complete the test. Furthermore, the GST protocol accepts each speed value with an accuracy of 3 out of 5. It was found to have worse results even at lower speeds, and it was observed that these conditions delayed the test time. Although our study concluded no statistically significant correlation between semicircular canal velocity functions and spatial abilities, it is considered a reference study for future cognitive vestibular studies.

Age was found to affect spatial learning and memory parameters significantly in our study. According to Williams et al. (2019), individuals between the ages of 40 and 55, which they classify as middle age, could show age-related spatial hypofunctions, particularly in their spatial memory abilities. Despite various evaluation methods, some studies have also shown that cognitive decline begins after middle age and becomes more evident by age 70 (Rönnlund et al., 2005). Still, they were unaffected before age 55–60 (Plassman et al., 1995). However, it was found that over 40 had a negative effect on spatial functions, the age factor was standardized with model analysis, and the impact of only visual motor functions was investigated and found to be significant. Therefore, the age-related findings are expected to contribute to the literature, particularly to research in this field.

The impacts of tobacco and glasses/contact lenses, in addition to age, have not yet been investigated in the studies in this field with VR systems. However, there are animal experiments on nicotine in the literature. It was emphasized that nicotine increased hippocampus activation in a study with rodents using the Morris Water Maze, and the authors suggested to employ as a treatment agent for age-related cognitive decline (King et al., 2011). On the other hand, another study indicates that nicotine has a negative impact since it impairs cardiovascular function and causes atrophy in the brain’s hippocampus region (Sacco et al., 2005). Furthermore, no studies were found in the literature regarding the impact of glasses and contact lens use on the vMWM test. However, our models indicate that the use of glasses has a negative effect on spatial memory parameters. Based on these findings, it is suggested that visual impairment itself does not adversely affect spatial abilities. Still, individuals wearing glasses may have experienced difficulties during the test, which could have negatively influenced the results. Further studies should focus on participants with glasses or lens use and patients with vestibulopathy, especially those accompanied by oscillopsia. This study has several limitations. Since we used a novel virtual approach to assess spatial skills, our comparisons between previous studies, especially in patients with vestibilopathies, lacked depth. Moreover, the duration of glasses usage was ignored. However, this could be important. Although we statistically performed model analysis for age, we still accept that the age range of the participants was broad. Further analysis should focus on participants with a specific age range.

## Conclusion

In conclusion, the effect of visual motor skills on spatial learning and spatial memory was investigated with a new test protocol in the vMWMT without disregarding the impact of various demographic factors. Spatial abilities in healthy adults between 18 and 55 were significantly affected by dynamic visual functions in the horizontal, vertical, and sagittal planes. Spatial learning and spatial memory have been the subject of numerous studies for many years, but interest in such topics has increased recently with the development of VR technology. The relationship between the vestibular system and the hippocampus was investigated from a new perspective in the present study. Modifying the vMWMT standard protocol and analyzing its correlations with other neuropsychological tests will also be crucial references in the literature for creating a current protocol.

## Data Availability

The raw data supporting the conclusions of this article will be made available by the authors, without undue reservation.
